# The Role of Anisotropy in Distinguishing Domination of Néel or Brownian Relaxation Contribution to Magnetic Inductive Heating: Orientations for Biomedical Applications

**DOI:** 10.3390/ma14081875

**Published:** 2021-04-09

**Authors:** Luu Huu Nguyen, Pham Thanh Phong, Pham Hong Nam, Do Hung Manh, Nguyen Thi Kim Thanh, Le Duc Tung, Nguyen Xuan Phuc

**Affiliations:** 1 Laboratory of Magnetism and Magnetic Materials, Advanced Institute of Materials Science, Ton Duc Thang University, Ho Chi Minh City 700000, Vietnam; phamthanhphong@tdtu.edu.vn; 2 Faculty of Applied Sciences, Ton Duc Thang University, Ho Chi Minh City 700000, Vietnam; 3 Institute of Materials Science, Vietnam Academy of Science and Technology, 18 Hoang Quoc Viet Street, Cau Giay District, Ha Noi 100000, Vietnam; namph.ims@gmail.com (P.H.N.); manhdh.ims@gmail.com (D.H.M.); 4 Graduate University of Science and Technology, 18 Hoang Quoc Viet Street, Cau Giay District, Ha Noi 100000, Vietnam; 5 Biophysics Group, Department of Physics and Astronomy, University College London, Gower Street, London WC1E 6BT, UK; 6 Healthcare Biomagnetic and Nanomaterials Laboratories, University College London, 21 Albemarle Street, London W1S 4BS, UK; 7 Duy Tan University, K7/25 Quang Trung Street, Da Nang City 550000, Vietnam; phucnx1949@gmail.com

**Keywords:** magnetic heating, Néel & Brownian relaxation, particle anisotropy, polydispersity, ferrofluid viscosity

## Abstract

Magnetic inductive heating (MIH) has been a topic of great interest because of its potential applications, especially in biomedicine. In this paper, the parameters characteristic for magnetic inductive heating power including maximum specific loss power (SLP_max_), optimal nanoparticle diameter (D_c_) and its width (ΔD_c_) are considered as being dependent on magnetic nanoparticle anisotropy (K). The calculated results suggest 3 different Néel-domination (N), overlapped Néel/Brownian (NB), and Brownian-domination (B) regions. The transition from NB- to B-region changes abruptly around critical anisotropy K_c_. For magnetic nanoparticles with low K (K < K_c_), the feature of SLP peaks is determined by a high value of D_c_ and small ΔD_c_ while those of the high K (K > K_c_) are opposite. The decreases of the SLP_max_ when increasing polydispersity and viscosity are characterized by different rates of d(SLP_max_)/dσ and d(SLP_max_)/dη depending on each domination region. The critical anisotropy K_c_ varies with the frequency of an alternating magnetic field. A possibility to improve heating power via increasing anisotropy is analyzed and deduced for Fe_3_O_4_ magnetic nanoparticles. For MIH application, the monodispersity requirement for magnetic nanoparticles in the B-region is less stringent, while materials in the N- and/or NB-regions are much more favorable in high viscous media. Experimental results on viscosity dependence of SLP for CoFe_2_O_4_ and MnFe_2_O_4_ ferrofluids are in good agreement with the calculations. These results indicated that magnetic nanoparticles in the N- and/or NB-regions are in general better for application in elevated viscosity media.

## 1. Introduction

MIH of magnetic nanoparticles (MNPs) while exposed to an alternating magnetic field (AFM) has attracted a lot of attention due to its potential applications in various domains, in particular cancer hyperthermia treatment and drug delivery. In cancer hyperthermia, it uses MIH on the principle that the cancer cells can be killed due to their higher thermal sensitivity than that of healthy ones [1,2,3,4,5,6,7,8,9,10,11]. For drug delivery, the MIH can be used to trigger remotely or on-site release of drug molecules being tagged to the MNPs [1,2,3,8,11,12,13]. Recently, new interesting biomedical applications were proposed including tuning the cellular gate for regulation of plasma glucose [14], biomaterials devitrification [15,16,17] and determination of MNPs accumulation in various organs [18]. In order to optimize the amount of MNPs used in the MIH, many studies focused on the conditions for maximizing heating efficiency, which is described theoretically by SLP or experimentally by specific absorption rate (SAR). The MIH power of MNPs has been known to consist of 3 main contributions: magnetization hysteresis, Néel relaxation generated by changing direction of the magnetic moments, and Brownian relaxation due to physical movement of MNPs. According to the linear response theory (LRT) [19,20], the Néel and Brownian relaxation processes are the main mechanisms to generate heat by the fluid containing superparamagnetic nanoparticles (NPs). Similar to other physical properties, the MIH power depends strongly on the diameter (D) of MNPs. Finding optimal particle diameter at which the SLP exhibits maximum value is frequently the purpose of both theoretical and experimental MNPs-MIH research.

Theoretical calculations indicated that, as a result of the competition between Néel and Brownian dissipation processes, the SLP versus D curve exhibits a peak shape with maximum value (SLP_max_) at a characteristic particle diameter D_c_ for different material parameters and field conditions [21,22,23,24,25,26]. From the studies of various Fe_3_O_4_ (FO) MNPs with different size at frequency f = 376 kHz, the maximum SLP_max_ was found for a 16 nm (size standard deviation, σ = 0.175) sample [27,28,29]; for iron oxide nanocubes at f = 520 kHz, it is 19 ± 3 nm [30,31]. An analysis of various data after taking a normalization of field factor gave an elevation of SLP in the diameter range from 14–18 nm [25]. For γ-Fe_2_O_3_, elevation of SLP for 5 sample sizes from 5.3 nm to 16.5 nm (σ = 0.19–0.43) is in agreement with the theoretical prediction for the peak appearance at D_c_ = 14.5 nm [22]. Although these results have confirmed the existence of optimal particle size D_c_ of MNPs in MIH, finding the value of D_c_ is not a simple task in experimental works because of the impact of other parameters such as the magnetic anisotropy, viscosity of fluid, and the size distribution.

Firstly, while the magnetic anisotropy of MNPs can be considered as a constant in theoretical works, it depends strongly on the particle size. Additionally, it was showed that the SLP_max_ and D_c_ decreases monotonically with increasing anisotropy [23,24]. It is noted that, due to the difference in morphology, the synthesized MNPs of the same material can have quite different polydispersity, anisotropy K values (e.g., FO MNPs having K ranging from about 20 to 550 kJ/m^3^ [32,33]) which can influence the SLP_max_ and D_c_ parameters. Secondly, the value of SLP_max_ was found to reduce with expansion of size distribution [22]. In practice, there will be some size distribution of the MNPs regardless of the synthesis method and producing MNPs with a sufficiently narrow size distribution is a difficult task. Thus far there have been few reports on experimental verification of calculated optimal D which would require the ability to synthesize MNPs with high monodispersity and to tune D precisely [22,25,28,34]. Thirdly, there might be some relationships between the value of D_c_ and SAR and ferrofluid viscosity. The size dependences of heating power with some variations of D and ferrofluid viscosity was studied experimentally and theoretically which showed for example for γ-Fe_2_O_3_ and CoFe_2_O_4_ MNPs SAR decreased about 20% and 80% with an increase of the viscosity, respectively [22]. To be able to obtain MNPs with optimal heating power, it is desirable to develop a complete correlation of SLP versus D and to consider impacts of different parameters, e.g., size distribution and shape of the NPs [20,21,22].

In this work, assessments of the impact of K on overall behavior of the heating power quantity have been systematically analyzed. The characteristics of SLP versus D for different MNPs have been studied by considering the impacts of two experimental parameters of the MNPs ferrofluid: σ and media viscosity (η). The different shapes of SLP(D) or the different rates of d(SLP_max_)/dσ and d(SLP_max_)/dη for FO MNPs with various anisotropy have been studied and explained as a result of the competition between Néel and Brownian dissipation processes. Here, the experimental results of MNPs of manganese ferrite (MFO), and cobalt ferrite (CFO) are considered as representative of low K (Néel domination) N type, and high K (Brownian domination) B type, which have confirmed the theoretical predictions.

## 2. Theoretical Basis

The SLP of the MNPs can be calculated as follows [4,20,21,22,23,24,25,26]:

(1) $$\mathrm{SLP}\;=\;\frac{P}{\phi \rho },$$

where Φ is the volume fraction (%), ρ the mean mass density (g/m^3^) and P the loss power density (W/m^3^). The calculations were carried out for particles with size standard deviation (or size polydispersity) parameter σ = 0–0.5, and viscosity η = 1–10 mPa·s, assuming the volume fraction Φ = 0.1% and the surface ligand layer thickness of 1 nm.

For the superparamagnetic NPs, it is generally known that Néel and Brownian relaxation losses mainly contribute to the MIH of materials. The Néel relaxation loss is originated from relaxation effects of magnetization in a magnetic field, the Brownian relaxation loss is due to the rotation of the NPs as a whole in a ferrofluid. In the LRT, χ is assumed to remain constant with increasing H. This approach was shown to be valid for small magnetic fields. Based on theoretical results, it was found that the condition of validity for the LRT is χ < 1. The dimensionless parameter, ξ, can be given by ref. [19]:

(2) $$\xi \;=\;\frac{\mu_{0}M_{s}VH}{k_{B}T},$$

Therefore, LRT based on Néel and Brownian relaxation losses is suitable for superparamagnetic NPs or H << H_C_ [19]. The correlation between SLP (based on LRT), the field amplitude (H), frequency (f) and the MNPs imaginary susceptibility (χ”) is ref. [20,24]:

(3) $$\mathrm{SLP}\;={\;\pi \mu }_{0}{\chi ”H}^{2}f,$$

with μ_0_ being the permeability of free space; where

(4) $$\chi ”\left(f\right)\;={\;\chi }_{0}\frac{f\tau }{1+{\left(f\tau \right)}^{2}},$$

The effective relaxation time (τ) can be determined from the compositional relaxation of Néel (τ_N_) and Brownian (τ_B_) processes [20,24]:

(5) $$\tau \;=\tau_{N}.\tau_{B}/\left(\tau_{N}+\tau_{B}\right),$$

with

(6) $$\tau_{N}\;=\;\frac{\sqrt{\pi }}{2}\tau_{0}\frac{\exp\left(KV/k_{B}T\right)}{\sqrt{\left(KV/k_{B}T\right)}},$$

(7) $$\tau_{B}\;=\;\frac{3{\eta V}_{H}}{k_{B}T},$$

where V and V_H_ are the volume of the core magnetic, and the whole capped particle, respectively.

For monodisperse superparamagnetic NPs (σ = 0), the value of SLP is calculated based on above equations. Considering the impact of particle size deviation (σ > 0), the mean loss power density can be described as ref. [20]:

(8) $$\bar{P}=\;\overset{\infty }{\underset{0}{\int }}Pg\left(D\right)dD,$$

with

(9) $$g\left(D\right)\;=\;\frac{1}{\sqrt{2\pi }\sigma D}\exp\left[-\frac{{\left(\ln\left(D/D_{0}\right)\right)}^{2}}{2\sigma^{2}}\right],$$

and g(D) the distribution function

(10) $$\overset{\infty }{\underset{0}{\int }}g\left(D\right)\mathrm{dD}\;=\;1,$$

## 3. Results and Discussion

### 3.1. Characteristics of Optimal Parameters in Domination Regions of Néel or Brownian Relaxations

**Figure 1:**
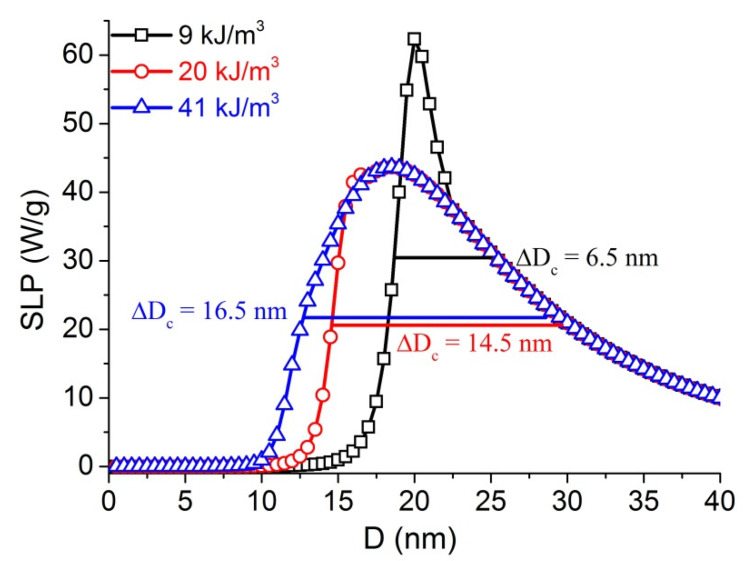
SLP versus D, calculated at f = 100 kHz, H = 5.18 kA/m, σ = 0, η = 1 mPa⋅s for monodispersive FO MNPs with 3 different K of 9, 20, and 41 kJ/m^3^. The bars denote the (full-width-half-maximum (FWHM)) ΔD_c_.

Magnetite is one of the extensively studied and important in a wide range of medical applications because of its biocompatibility. The anisotropy value of FO MNPs was reported between a few tens to higher than 500 kJ/m^3^ [32,33]. The calculations are, therefore, made for different K up to 50 kJ/m^3^ and the results of SLP versus particle diameter at K equal to 9 kJ/m^3^, 20 kJ/m^3^, 41 kJ/m^3^ are presented in Figure 1.

The typical peak behaviors of the calculated SLP against D obtained for FO MNPs can be seen in Figure 1. Here, the difference in the SLP (D) graph shape of FO MNPs depends on the value of K [20,23]. We have introduced full-width-half-maximum (FWHM) ΔD_c_ for describing the peaks. The D_c_, ΔD_c_ and SLP_max_ obtained at f = 100 kHz, H = 5.18 kA/m (65 Oe), σ = 0, η = 1 mPa⋅s for monodisperse FO ferrofluids with different anisotropy K are listed in Table 1.

The results showed that the high K (50 kJ/m^3^) FO sample (18.5 nm) has much smaller value of D_c_ compared with that of the low K (3 kJ/m^3^) one (28.5 nm). In contrast, there is a large increase in the value of FWHM ΔD_c_ when the value of K goes from 3 kJ/m^3^ (3.5 nm) to 50 kJ/m^3^ (16.5 nm). The fact that the SLP peak is narrow for low K but broad for high K can be attributed to Néel or Brownian domination, respectively [9,20,22,24].

Based on Equation (5), it is clear that the effective relaxation time is dominated by the shorter of the two components. For example, A. E. Deatsch et al. found that the Brownian relaxation becomes more significant at 13 nm for high anisotropy particles (40 kJ/m^3^) [25]. We calculated the value of relaxation times at D_c_ for all samples. As can be seen in Table 1, the value of effective relaxation time is approximately equal to the Brownian relaxation time at D_c_ when the value of K is higher than 19 kJ/m^3^. The calculations indicated that the Brownian relaxation dominated at D_c_ when the value of K is higher than 19 kJ/m^3^. Therefore, the difference in peak behaviors of the SLP against D depends on the domination of Néel or Brownian relaxations. As can be seen in Table 1, the value of D_c_ changes with an increase of K from 3 kJ/m^3^ to 20 kJ/m^3^, because the Néel relaxation loss still affects this parameter. When the value of K for FO is higher than 20 kJ/m^3^, D_c_ and SLP_max_ are unchanged due to the domination of Brownian relaxation loss. Therefore, it changes abruptly at some critical anisotropy K_c_ = 20 kJ/m^3^.

**Figure 2:**
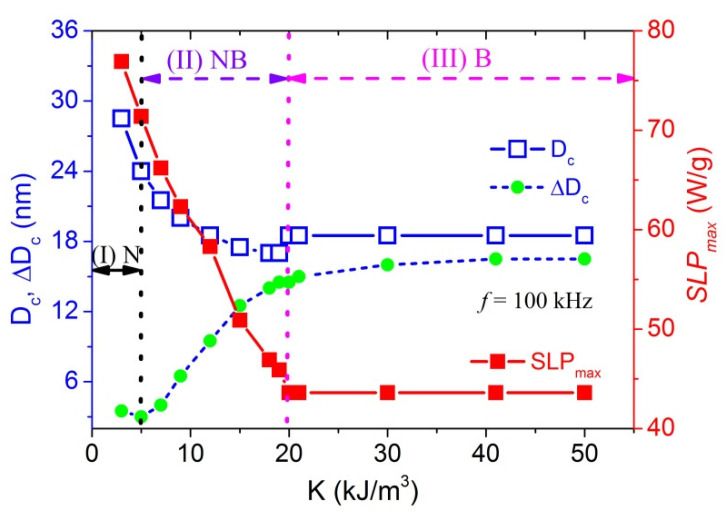
Changes of characteristic parameters, SLP_max_, D_c_ and ΔD_c_ from Néel-domination to Brownian-domination region calculated for frequency f = 100 kHz, viscosity η = 1 mPa⋅s.

Changes of characteristic parameters, SLP_max_, D_c_ and ΔD_c_ as a function of K for f = 100 kHz, η = 1 mPa⋅s are presented in Figure 2, where it suggests 3 different regions. In the lowest K (region I: K ≤ 5 kJ/m^3^): SLP_max_, D_c_ and ΔD_c_ decrease with increasing K. The middle region (region II: 5 kJ/m^3^ ≤ K ≤ 20 kJ/m^3^) is characterized by a tendency of the decrease of D_c_ to slow down while ΔD_c_ starts to increase with increasing K. In the high K region (region III: K > 20 kJ/m^3^), all the 3 parameters become almost constant. Relating to the dissipation mechanisms, the regions I, II and III can be correspondingly assigned as Néel-domination (N-region), Néel/Brownian overlap (NB-region) and Brownian-domination (B-region). While the transition from N- to NB-region is quite smooth, the transition to the B region is distinctly of the first order and in case of FO MNPs it corresponds to a critical anisotropy value K_c_ of about 20 kJ/m^3^.

### 3.2. Dependence of Critical Anisotropy K_c_ on Frequency (f)

Based on LRT, the value of SLP reaches the maximum SLP_max_ at the optimal particle size D_c_ when the condition ωτ = 1 is satisfied. Therefore, the anisotropy boundary of the transition from NB- to B-region might depend on the frequency of AMF.

**Figure 3:**
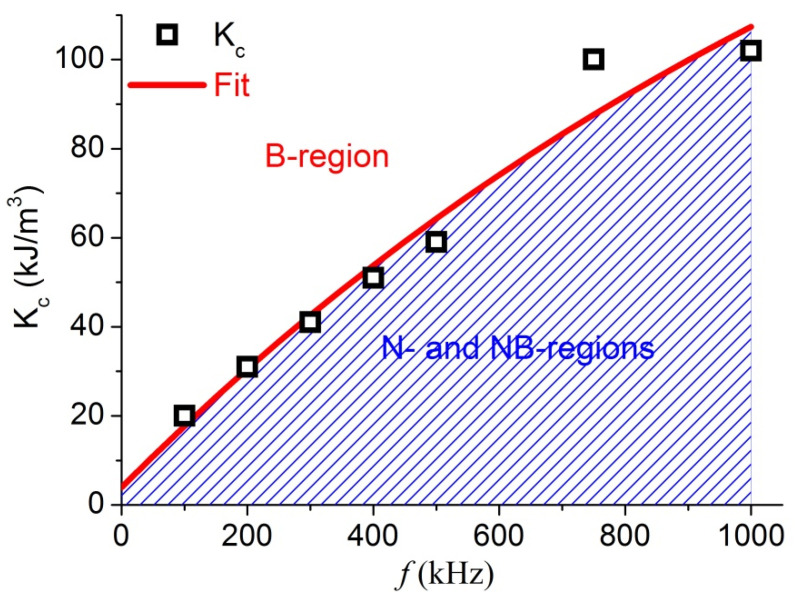
K_c_ versus f at η = 1 mPa⋅s, the fitting gave R^2^ = 0.94121.

Based on the data of FO MNPs at η = 1 mPa⋅s for the expanded K range and at various frequencies, the SLP was calculated and results of D_c_ versus K are presented in Figure S1a. For frequencies up to 1000 kHz can be seen an abrupt change of D_c_ at K_c_ associated with the transition to the Brownian domination region (for example with f = 500 kHz, the value of K_c_ is about 60 kJ/m^3^, and the three N, NB and B regions are presented in Figure S1b. The dependence of the critical anisotropy K_c_ on the field frequency is presented in Figure 3, which follows the function:

(11) $$K_{c}\left(f\right)\;=\;A\left(1-e^{-B\times (f+f_{0})}\right),$$

where A = 214.63 kJ/m^3^, B = 0.0000814 ms, and f_0_ = 81.27 kHz are fitting constants.

The values of critical anisotropy K_c_ at different frequencies for fluids with various viscosities are listed in Table 2. For MFO fluid with K = 3 kJ/m^3^, the Brownian relaxation dominates when η = 1 mPa⋅s and f = 100 kHz (low frequency) but it becomes the Néel relaxation domination when η ≥ 1 mPa⋅s or f ≥ 100 kHz. For CFO fluid, the Néel relaxation dominates when η ≥ 4 mPa⋅s or f ≥ 500 kHz, but it is the Brownian relaxation domination when η ≤ 2 mPa⋅s or f ≤ 1000 kHz.

### 3.3. Orientations to Choose Proper Regions in Biomedical Applications

**Figure 4:**
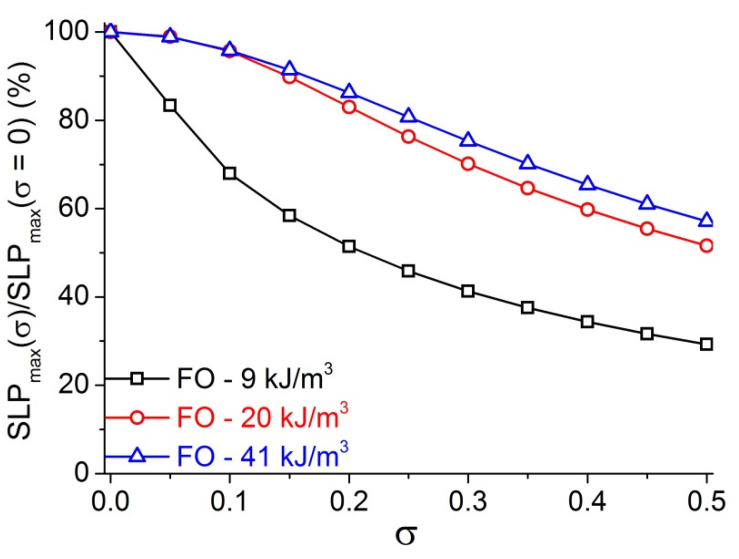
SLP_max_(σ)/SLP_max_(σ = 0) calculated at f = 100 kHz, H = 5.18 kA/m, η = 1 mPa⋅s for FO samples with K = 9, 20, and 41 kJ/m^3^.

For biomedical applications, the size distribution and the viscosity of ferrofluid must be considered. In the above calculation of the relaxation times and MIH power of MNPs, the particles were assumed to have the same size, in other words the MNPs are monodispersive with σ = 0. However, in reality, this assumption cannot be satisfied because usually there would exist some size distribution of the NPs regardless of the synthetic method used. In practice, producing monodisperse MNPs (σ = 0) is almost impossible task. For example, J. P. Fortin et al. synthesized 16.5 nm γ-Fe_2_O_3_ with σ = 0.43, which could be reduced to 0.19 (d = 5.3 nm) after size sorting by successive phase separations [22]. M.P. Pileni et al. synthesized 2–5 nm CoFe_2_O_4_ with 30% polydispersity in size distribution by using functionalized surfactants [35]. When A. K. Gupta et al. compared the different characteristic features of the iron oxide NPs fabricated through different methods, they were always polydisperse NPs with narrow or broad size distributions [3]. Therefore, the assumption (σ = 0) appears not to be realized. In fact, in the synthesis of MNPs using seed mediated-growth or size sorting route, the common value of σ was reported in between 0.3–0.5 but sometime it can reduce to 0.15–0.2 [2,22,36,37]. The polydispersity characterized by the σ of MNPs can give rise to the reduction of SLP [20]. For γ-Fe_2_O_3_ MNPs, σ in the range of 0.08–0.15 could result in a decrease of SLP_max_ to half of the monodispersive value [22,24]. Calculations were carried out for various anisotropy (K) and standard deviation (σ) of FO MNPs at f = 100 kHz, H = 5.18 kA/m, η = 1 mPa⋅s and the results of relative SLP_max_(σ)/SLP_max_(σ = 0) are represented in Figure 4.

The change of heating power behavior with varying K has been reflected clearly also in the dependence of relative SLP on size standard variation. It can be seen that the domination region strongly influences the behavior of SLP besides material composition and particle size. Our results in Figure 4 indicate that, in all the curves, the SLP_max_ decreases with increasing σ and there is a clear trend of increasing relative SLP_max_ when the value of K changes from the NB-region (9 kJ/m^3^) to K_c_ (20 kJ/m^3^) and then the B-region (41 kJ/m^3^). In other words, for the similar polydispersity degree σ, the MNPs in the B-region would have higher heating power than that of those in the NB-region. These results indicated that the monodispersity requirement for getting the same heating power is much less strict for the MNPs in B-region as compared with the case of MNPs in N- or NB-region. Quantitatively, the polydispersity-caused SLP reduction for iso-dispersity of σ = 0.2 FO MNPs and σ_50_ (defined as at the position of ½ SLP_max_) with various anisotropies at f = 100 kHz, H = 5.18 kA/m, η = 1 mPa⋅s are listed in Table 3. The parameter σ_50_ is seen to increase from 0.21 to 0.58 with K from 9 to 32 kJ/m^3^, then becomes constant for K ≥ 36 kJ/m^3^ indicating that the monodispersity requirement for obtaining the same heating power is much less strict for the high K as compared with the case of low K MNPs. The results in Table 3 also show that the polydispersity-caused SLP reduction decreased with an increase of magnetic anisotropy. From the slope of the SLP graph in Figure S2, it is estimated that an increase of K by about 4 times can result in saving as much as 35% of the heating power. These results seem to be consistent with the observation on cubic-shaped FO MNPs of about 20 nm (K = 180 kJ/m^3^) having higher heating power than spherical ones (K = 20 kJ/m^3^) [32]. The improvement of heating power by increasing K is an interesting topic and it has been recently reported for different materials, while the elevation of K was possible by various approaches including surface morphology, exchange-bias or stacking of particles in chains [32,33,36,37,38,39].

**Figure 5:**
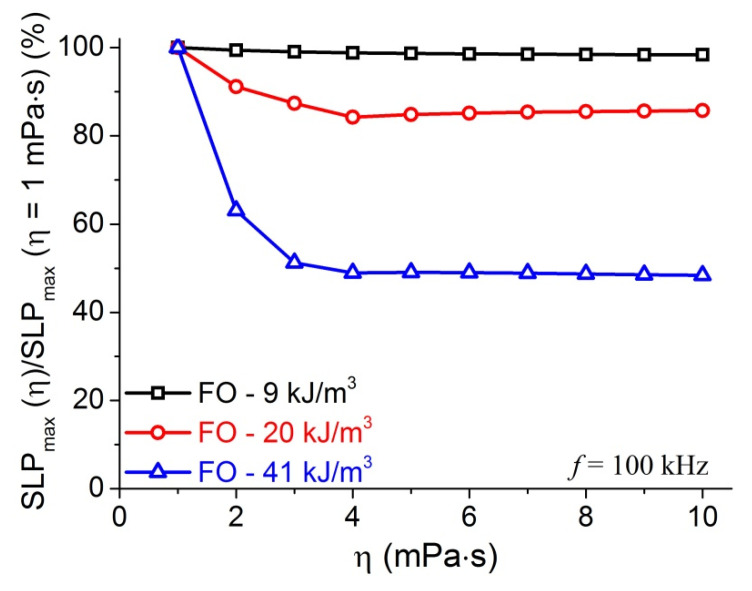
SLP_max_(η)/SLP_max_(η = 1 mPa⋅s) calculated at f = 100 kHz, H = 5.18 kA/m for FO samples with K = 9, 20, and 41 kJ/m^3^.

The medium in which MNPs are dispersed also can affect the heating efficacy of MNPs. Based on Equations (3)–(7), Rosensweig’s calculations demonstrated a dependence of the heating rate ΔT/Δt on the medium viscosity at 3 different frequencies [20]. We performed systematic calculations of SLP_max_ in the viscosity range from 1 mPa⋅s to 10 mPa⋅s for MNPs with various K. The results of the relative SLP_max_(η)/SLP_max_(η = 1 mPa⋅s) for the fluid of FO MNPs are presented in Figure 5. As can be seen in Figure 5, for FO MNPs with the value of K in NB-region (9 kJ/m^3^), SLP_max_ decreases very little with increasing η. In contrast, there is a large decrease in the SLP_max_ when the value of η is going from 1 kJ/m^3^ to 4 mPa⋅s for 20 kJ/m^3^, and 41 kJ/m^3^ FO MNPs. It is noted that the value of critical anisotropy at 4 mPa⋅s for the field frequency of 100 kHz is 60 kJ/m^3^ (Table 2). Therefore, the decrease of SLP_max_ with increasing η might occur when the value of anisotropy is smaller than K_c_. In other words, this behavior happens when the K of FO MNPs is in the B-region. These calculations indicated that the FO MNPs with the K in the N- or NB-regions are much better for MIH applications in highly viscous conditions. The observation of weak and strong decrease of the heating power with increasing viscosity, will be discussed as compared with experimental results in the following section.

### 3.4. A Comparison with Experimental Results

To compare the results from the experiments with the theoretical ones in a simple way, we have investigated SLP of the same MNPs but varying viscosity. Previously we reported the SAR measurements carried out at the field of 178 kHz and 5.18 kA/m (65 Oe) for bare CFO and MFO MNPs suspended in water fluid with added agar forming media with viscosity up to about 2 mPa⋅s, and observed strong and weak decrease of SAR with an increase of η, respectively for CFO and MFO, which is in agreement with the LRT prediction [40]. For comparing the results from the experiments with the theoretical ones, we studied two ferrofluids of 20 nm low-K MnFe_2_O_4_ and 15 nm high-K CoFe_2_O_4_ MNPs coated with chitosan. They were prepared by co-precipitation. To create the magnetic fluids with different viscosity, the MNPs suspension was mixed with various agar concentrations. The value of viscosity extended up to about 8 mPa⋅s by adding more agar amounts. The fabrication of samples with various viscosities as well as the method of viscosity measurement were described earlier [40] as were the experimental details of the synthesis and the coating procedure by chitosan [41]. As discussed in our previous reports, chitosan has been widely used in biomedicine due to its biocompatibility and biodegradability [41]. Based on the measured magnetization curves shown in Figure S3, the thickness of chitosan coating layer was estimated to be around 4 nm. The value of M_S_, H_C_ and K_eff_ for MFO and CFO NPs (shown details in Supplementary Section S3) are presented in Table 4.

In addition to the field of 178 kHz, 5.18 kA/m (provided by a commercial generator RDO HFI 5 kW [40]), the calorimetric measurements were additionally conducted at two fields of the same amplitude of 15.9 kA/m (200 Oe) with frequencies of 340 kHz and 450 kHz (provided by a commercial generator UHF-20A [42]), to seek evidence of SAR behavior changing with increasing field frequency. The temperature elevation with time, T(t), was monitored by using the same commercial optical sensor (GaAs-Opsens) for both MIH sets up.

**Figure 6:**
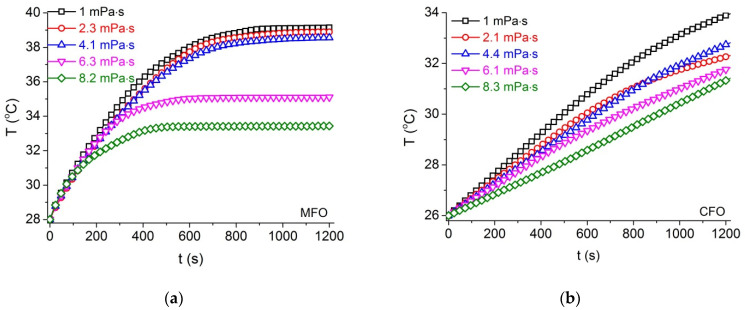
Hyperthermia curves measured at field frequency f = 178 kHz, H = 5.18 kA/m for (**a**) MFO and (**b**) CFO ferrofluids of various viscosities.

The heating curves, measured at the lower field, for the two series of MFO and CFO samples in solutions of various agar concentrations are presented in Figure 6. The values of ${\mathrm{SAR}}_{\exp}$ were calculated from the fitting of the temperature rising curves T(t) for the whole time range following the method described in reference [40].

In order to compare the experimental and theoretical SAR, we calculated the Néel-Brownian relaxation experimental ${\mathrm{SAR}}_{\exp}^{\mathrm{LRT}}$ by using the Equation:

(12) $${\mathrm{SAR}}_{\exp}^{\mathrm{LRT}}\;={\;\mathrm{SAR}}_{\exp}-{\mathrm{SAR}}_{\exp}^{\mathrm{hys}},$$

where ${\mathrm{SAR}}_{\exp}$ is the SAR contribution by hysteresis mechanism, whose estimation is described in Supplementary Section S3, similarly to the approach reported in [42]. The results of calculated ${\mathrm{SAR}}_{\exp}^{\mathrm{LRT}}$ obtained from the measurements at 340 kHz and 450 kHz. They are gathered in Supplementary Section S4. As can be seen, for the MFO samples with K = 11 kJ/m^3^ and H_c_ were close to or smaller than the AFM amplitudes, the contribution of ${\mathrm{SAR}}_{\exp}^{\mathrm{hys}}$ is less than 2.5% of the whole heating power. However, for the CFO samples with K = 62 kJ/m^3^ and H_c_ much larger than the field amplitude used, the maximum ${\mathrm{SAR}}_{\exp}^{\mathrm{hys}}$ contributions were 17%, 16% and 9% for 178, 340 and 145 kHz, respectively.

**Figure 7:**
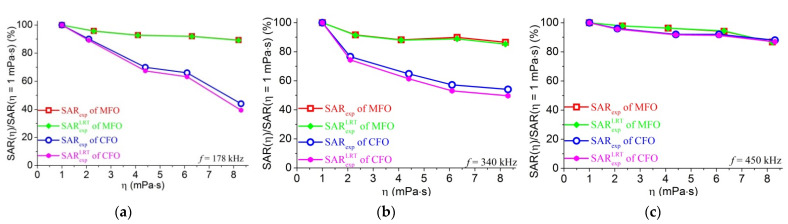
Relative ${\mathrm{SAR}}_{\exp}$ and ${\mathrm{SAR}}_{\exp}^{\mathrm{LRT}}$ measured at field of (**a**) 5.18 kA/m, 178 kHz; (**b**) 15.9 kA/m, 340 kHz; and (**c**) 15.9 kA/m, 450 kHz.

In order to discuss about the impact of AFM frequency on the SAR characteristics relevant to the relaxation mechanisms, we calculated the ratio of SAR(η)/SAR(η = 1 mPa⋅s) for all the MFO and CFO samples of various viscosities measured at the 3 frequencies (see also Tables S1–S3), the dependences of which are shown in Figure 7.

Firstly, it can be noted that the decrease of SAR with increasing viscosity is experimentally observed in all the cases, so that the impact of the hysteresis contribution is insignificant.

Secondly, Figure 7a,b suggest that the strong and weak decrease of SAR with increasing viscosity, depicted respectively for CFO and MFO, confirm our previous experiment reported for η ≤ 2.12 mPa⋅s [40] and the agreement with the LRT theory prediction shown in Figure 5. While such a behavior is natural for the case of the field (178 kHz, 65 Oe) with the parameter ξ of about 0.7, it is worth noting that such behavior ‘survived’ to the field of 340 kHz and 15.9 kA/m with ξ of about 2. The trend of decreasing SAR with increasing viscosity η in our experiments is also in agreement with the observation for γ-Fe_2_O_3_ (D = 7.1 nm, σ = 0.37) and CoFe_2_O_4_ (D = 9.7 nm, σ = 0.35) MNPs where SAR decreased, respectively, about 20% and 80% when the viscosity increased from 0.75 to 335 mPa⋅s [22]. In fact, it was found that SLP value from MNPs dispersed in water is higher than in glycerol for the same type of MNPs, MNP dose, and AMF condition [43]. A similar phenomenon was observed in the cellular environment as well, in which the measured SLP value decreased by half associated with attenuation of the Brownian relaxation in cellular conditions [44]. Thirdly, while the MFO ferrofluids remain almost not impacted by the field frequency used, it is interesting to observe for CFO samples a clear shift from high K toward low-K characteristic behavior when the used field frequency increases from 178 kHz to 450 kHz. In particular, Figure 7c for the case of f = 450 kHz indicates that the relative SAR versus η for the CFO MNPs approaches to the type of very weak decrease of the heating power with increasing the media viscosities, similarly to that of MFO MNPs. This observation is in good agreement with the scheme shown in Figure S7, according to which the MFO MNPs of K = 11 kJ/m^3^ are predicted to exhibit the SAR contributed mainly from the Neel or overlapped Neel-Brownian relaxation losses depending not on the used field frequency, and the dissipation mechanism contributing to the SAR for the CFO MNPs with K = 62 kJ/m^3^ belongs to the pure Brownian region for AMF with f below about 460 kHz and changes to the Neel-Brown or Neel type above this frequency.

## 4. Conclusions

In summary, in addition to the peak position D_c_ and its amplitude SLP_max_, we have introduced a peak width ΔD_c_ and performed systematic analyses of the three parameters against the magnetic anisotropy of MNPs, fluid viscosities and the frequency of the alternating magnetic field. High K MNPs have been shown to exhibit domination of Brownian loss while it becomes that of Néel loss in low K ones. To the best of our knowledge, our results have demonstrated for the first time the transition from Néel to Brownian loss region not to occur in a continuous way, but at critical anisotropy K_c_ which increases with the frequency of the alternating magnetic field and the viscosity of the ferrofluid. We have pointed out that, for a given material, fabrication of MNPs with higher anisotropy up to K_c_ can improve the heating power as much as 35% thanks to the offsetting of the polydispersity-caused reduction. From the calculation and experimental results, it appears that low K MNPs are in general better for MIH applications in highly viscous conditions than those of high K ones.

## Figures and Tables

**Table 1:** D_c_, ΔD_c_, SLP_max_, τ_N_, τ_B_, and τ obtained for monodisperse FO ferrofluids with different anisotropy constant K. (f = 100 kHz, H = 5.18 kA/m, σ = 0, η = 1 mPa⋅s).

K (kJ/m^3^)	D_c_ (nm)	ΔD_c_ (nm)	SLP_max_ (W/g)	τ_N_ (s)	τ_B_ (s)	τ (s)
3	28.5	3.5	76.9	$1.95\times 10^{-6}$	$10.77\times 10^{-6}$	$1.65\times 10^{-6}$
5	24	3	71.4	$1.87\times 10^{-6}$	$6.67\times 10^{-6}$	$1.47\times 10^{-6}$
7	21.5	4	66.2	$1.98\times 10^{-6}$	$4.92\times 10^{-6}$	$1.42\times 10^{-6}$
9	20	6.5	62.3	$2.65\times 10^{-6}$	$4\times 10^{-6}$	$1.6\times 10^{-6}$
12	18.5	9.5	58.3	$4.26\times 10^{-6}$	$3.27\times 10^{-6}$	$1.85\times 10^{-6}$
15	17.5	12.5	50.9	$3.24\times 10^{-6}$	$2.81\times 10^{-6}$	$2.02\times 10^{-6}$
19	17	14	46.9	$3.46\times 10^{-6}$	$2.6\times 10^{-6}$	$1.52\times 10^{-6}$
20	17	14.5	45.9	$2\times 10^{-3}$	$2.6\times 10^{-6}$	$2.6\times 10^{-6}$
21	18.5	14.5	43.6	$4\times 10^{-3}$	$3.27\times 10^{-6}$	$3.27\times 10^{-6}$
30	18.5	15	43.6	4.903	$3.27\times 10^{-6}$	$3.27\times 10^{-6}$
41	18.5	16	43.6	$2.8\times 10^{4}$	$3.27\times 10^{-6}$	$3.27\times 10^{-6}$
50	18.5	16.5	43.6	$3.4\times 10^{7}$	$3.27\times 10^{-6}$	$3.27\times 10^{-6}$

**Table 2:** Values of K_c_ at different frequencies for fluids with various viscosities (η (mPa⋅s)).

f (kHz)	K_c_ (kJ/m^3^)				
	η = 1	η = 2	η = 4	η = 6	η = 8
100	20	33	60	83	102
200	31	56	103	147	205
300	41	74	141	207	261
400	51	95	188	252	322
500	59	112	227	308	399
750	100	153	292	>400	>400
1000	102	205	364	>400	>400

**Table 3:** Polydispersity-caused SLP reduction for iso-dispersity σ = 0.2 FO MNPs and σ_50,_ defined as at the position of ½ SLP_max_, with various anisotropy K (f = 100 kHz, H = 5.18 kA/m, η = 1 mPa⋅s).

K (kJ/m^3^)	SLP Reduction (%)	σ_50_
9	48.6	0.21
12	38.8	0.3
16	25.3	0.43
20	17	0.52
24	15.6	0.54
28	14.8	0.57
32	14.3	0.58
36	13.9	0.59
40	13.9	0.59

**Table 4:** Values of K_c_ at different frequencies for fluids with various viscosities (η (mPa⋅s)).

Sample	M_S_ (emu/g)	H_C_ (Oe)	K_eff_ (kJ/m^3^)
MFO (uncoated)	60.9	72	-
MFO (coated)	51.9	72	11
CFO (uncoated)	67.2	875	-
CFO (coated)	57.4	875	62

## Data Availability

Data is contained within the article or Supplementary Materials.

## Supplementary Materials

The following are available online at https://www.mdpi.com/article/10.3390/ma14081875/s1, Figure S1: (a) D_c_ versus K for various AMF frequencies. (b) The width ΔD_c_, and Dc versus K at f = 500 kHz showing 3 characteristic N, NB and B regions; Figure S2: Polydispersity-caused SLP reduction calculated at f = 100 kHz for iso-dispersity σ = 0.2 FO MNPs as a function of anisotropy K, Figure S3: Magnetization curves measured for as-synthesized and chitosan coated (a) MFO, and (b) CFO MNPs; Figure S4: The initial magnetization curves of MFO and CFO MNPs. The solid lines represent the fitting curve assuming “the law of approach to saturation”; Figure S5: Hyperthermia curves measured at fields of frequency f = 340 kHz, H = 15.9 kA/m (200 Oe) for (a) MFO and (b) CFO ferrofluids of various viscosities; Figure S6: Hyperthermia curves measured at fields of frequency f = 450 kHz, H = 15.9 kA/m for (a) MFO and (b) CFO ferrofluids of various viscosities; Figure S7: Illustration scheme for the MIH experiments for CFO and MFO MNPs, Table S1: Values of ${\mathrm{SAR}}_{\exp}$, ${\mathrm{SAR}}_{\exp}^{\mathrm{hys}}$, ${\mathrm{SAR}}_{\exp}^{\mathrm{LRT}}$, and $\frac{{\mathrm{SAR}}_{\exp}^{\mathrm{LRT}}}{{\mathrm{SAR}}_{\exp}^{\mathrm{LRT}}\;\left(\eta =1\;\mathrm{mPa}\cdot s\right)}$ at 5.18 kA/m, 178 kHz; Table S2: Values of ${\mathrm{SAR}}_{\exp}$, ${\mathrm{SAR}}_{\exp}^{\mathrm{hys}}$, ${\mathrm{SAR}}_{\exp}^{\mathrm{LRT}}$, and ${\mathrm{SLP}}^{\mathrm{LRT}}$ at 15.9 kA/m, 340 kHz; Table S3: Values of ${\mathrm{SAR}}_{\exp}$, ${\mathrm{SAR}}_{\exp}^{\mathrm{hys}}$, ${\mathrm{SAR}}_{\exp}^{\mathrm{LRT}}$, and ${\mathrm{SLP}}^{\mathrm{LRT}}$ at 15.9 kA/m, 450 kHz.
