# Supplementary material for: The Role of Anisotropy in Distinguishing Domination of Néel or Brownian Relaxation Contribution to Magnetic Inductive Heating: Orientations for Biomedical Applications
Source: Materials (Basel). 2021 Apr 9;14(8):1875. doi: 10.3390/ma14081875 (PMC8070233; doi:10.3390/ma14081875)
Supplement: Supplementary file 1 [file materials-14-01875-s001.pdf]

materials

SUPPLEMENTARY

The Role of Anisotropy in Distinguishing Domination of Néel or Brownian Relaxation Contribution to Magnetic Inductive Heating: Orientations for Biomedical Applications

Luu Huu Nguyen [1,2,*], Pham Thanh Phong [1,2], Pham Hong Nam [3,4], Do Hung Manh [3,4], Nguyen Thi Kim Thanh [5,6,*], Le Duc Tung [5,6,*] and Nguyen Xuan Phuc [7]

[1] Laboratory of Magnetism and Magnetic Materials, Advanced Institute of Materials Science, Ton Duc Thang University, 700000 Ho Chi Minh City, Vietnam; phamthanhphong@tdtu.edu.vn
[2] Faculty of Applied Sciences, Ton Duc Thang University, 700000 Ho Chi Minh City, Vietnam
[3] Institute of Materials Science, Vietnam Academy of Science and Technology, 18 Hoang Quoc Viet Street, Cau Giay District, 100000 Ha Noi, Vietnam; namph.ims@gmail.com (P.H.N.); manhdh.ims@gmail.com (D.H.M.)
[4] Graduate University of Science and Technology, 18 Hoang Quoc Viet Street, Cau Giay District, 100000 Ha Noi, Vietnam
[5] Biophysics Group, Department of Physics and Astronomy, University College London, Gower Street, London WC1E 6BT, UK
[6] Healthcare Biomagnetic and Nanomaterials Laboratories, University College London, 21 Albemarle Street, London W1S 4BS, UK
[7] Duy Tan University, K7/25 Quang Trung Street, 550000 Da Nang City, Vietnam; phucnx1949@gmail.com
* Correspondence: luuhuunguyen@tdtu.edu.vn (L.H.N.); ntk.thanh@ucl.ac.uk (N.T.K.T.); t.le@ucl.ac.uk (L.D.T.)

Supplementary Section 1: D_c and ΔD_c versus K and Their Region Characteristics

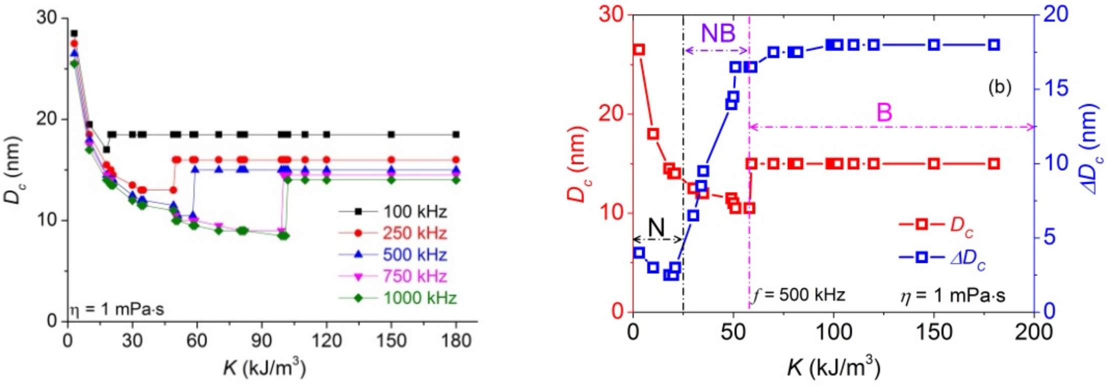

(**a**) (**b**)

Figure S1. (**a**) D_c versus K for various AMF frequencies. (**b**) The width ΔD_c, and Dc versus K at f = 500 kHz showing 3 characteristic N, NB and B regions.

Supplementary Section 2: Polydispersity-Caused SLP Reduction Calculated for MNPs of the Same Parameter σ but Various Anisotropy K

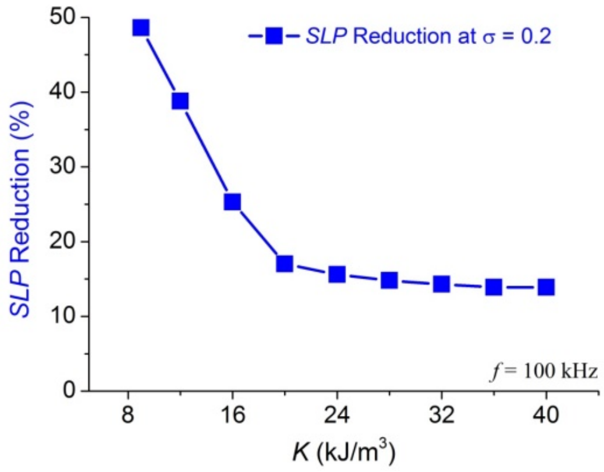

Figure S2. Polydispersity-caused SLP reduction calculated at f = 100 kHz for iso-dispersity σ = 0.2 FO MNPs as a function of anisotropy K.

Supplementary Section 3: Magnetization Curves of $MnFe_2O_4$ (MFO) and $CoFe_2O_4$ (CFO) MNPs

The saturation magnetization of the samples at room temperature was measured under the highest magnetic field of 876 kA/m (~11 kOe) using a home-made vibrating sample magnetometer (VSM).

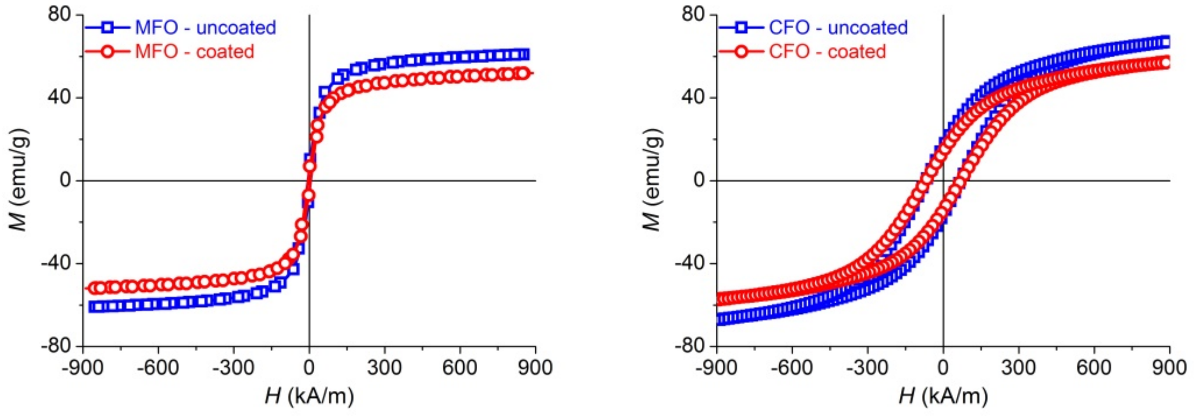

(**a**) (**b**)

Figure S3. Magnetization curves measured for as-synthesized and chitosan coated (**a**) MFO, and (**b**) CFO MNPs.

In order to calculate the Keff values for MFO and CFO nanoparticles, we adopted the method described in ref. [42]. Briefly, the experimental data of initial magnetization curves were fitted under "the law of approach to saturation" (Figure S4):

$$M(H) = M_s\left(1 - \frac{a}{H} - \frac{b}{H^2} - \cdots\right) + \chi_p H \quad , \tag{S1}$$

where χ_P is the high field differential susceptibility and a, b free parameters.

The effective magnetic anisotropy can be calculated by Equation:

$$b = \frac{4K_{eff}^2}{15M_S^2}, \tag{S2}$$

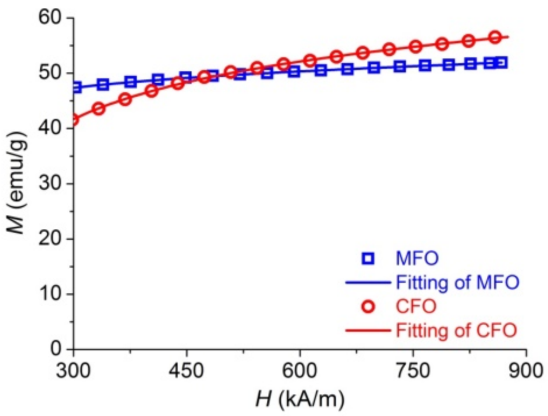

Figure S4. The initial magnetization curves of MFO and CFO MNPs. The solid lines represent the fitting curve assuming "the law of approach to saturation".

Supplementary Section 4: Detailed Calculation of $\mathrm{SAR}_{\mathrm{exp}}^{\mathrm{hys}}$

The hysteresis loss power of MNPs can be written as ref. [23]:

$$P^{hys} = (4\mu_0 M_S H_a)f, \tag{S3}$$

For the case of randomly oriented MNPs, it was indicated that the value of H_a is given as ref. [23]:

$$\mu_0 H_a = 0.48\mu_0 H_K(b - \kappa^n), \tag{S4}$$

in which, $b = 0.9$, $n = 1$ and H_K ($= \frac{2K_{eff}}{\mu_0 M_S}$) is the coercive field.

If $Ha \geq H_0$, the hysteresis loss power of MNPs was calculated with the value of H_0 [23].

Therefore, the $\mathrm{SAR}_{\mathrm{exp}}^{\mathrm{hys}}$ of the MNPs can be calculated as follows:

$$\mathrm{SAR}_{\mathrm{exp}}^{\mathrm{hys}} = \frac{P^{hys}}{\phi\rho}, \quad \text{(S5)}$$

Supplementary Section 5: Results of SAR MFO and CFO Nanoparticles

Table S1. Values of $\mathrm{SAR}_{\mathrm{exp}}$, $\mathrm{SAR}_{\mathrm{exp}}^{\mathrm{hys}}$, $\mathrm{SAR}_{\mathrm{exp}}^{\mathrm{LRT}}$, and $\frac{\mathrm{SAR}_{\mathrm{exp}}^{\mathrm{LRT}}}{\mathrm{SAR}_{\mathrm{exp}}^{\mathrm{LRT}}(\eta = 1\ \mathrm{mPa{\cdot}s})}$ at 5.18 kA/m, 178 kHz.

**Sample**	**Viscosity (mPa·s)**	$\mathbf{SAR_{exp}}$ **(W/g)**	$\mathbf{SAR_{exp}^{hys}}$ **(W/g)**	$\mathbf{SAR_{exp}^{LRT}}$ **(W/g)**	$\frac{\mathbf{SAR_{exp}^{LRT}}}{\mathbf{SAR_{exp}^{LRT}(\eta = 1mPa \cdot s)}}$ **(%)**
MFO	1	77.7	1.5	76.2	100
	2.3	74.4	1.5	72.9	96
	4.1	72.1	1.5	70.6	93
	6.3	71.5	1.5	70	92
	8.2	69.4	1.5	67.9	89
CFO	1	20.9	1.6	19.3	100
	2.1	18.8	1.6	17.2	89
	4.4	14.6	1.6	13	67
	6.1	13.8	1.6	12.2	63
	8.3	9.2	1.6	7.6	39

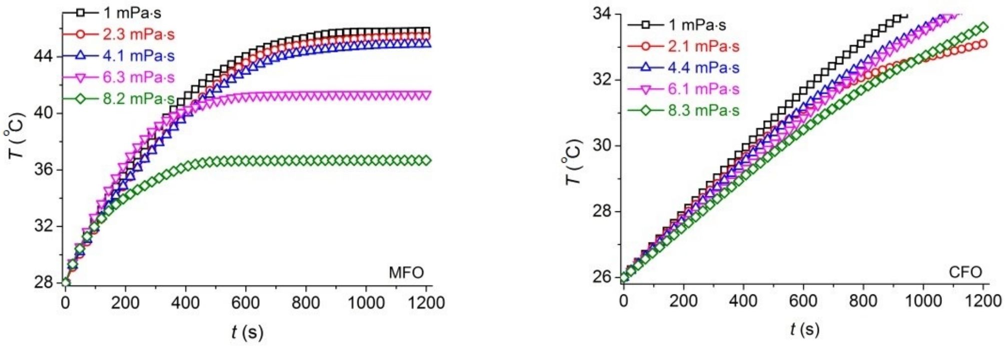

(**a**) (**b**)

Figure S5. Hyperthermia curves measured at fields of frequency f = 340 kHz, H = 15.9 kA/m (200 Oe) for **(a)** MFO and **(b)** CFO ferrofluids of various viscosities.

Table S2. Values of SAR_{exp}, SAR_{exp}^{hys}, SAR_{exp}^{LRT}, and SLP^{LRT} at 15.9 kA/m, 340 kHz

Sample	Viscosity (mPa·s)	SAR_{exp} (W/g)	SAR_{exp}^{hys} (W/g)	SAR_{exp}^{LRT} (W/g)	$\frac{SAR_{exp}^{LRT}}{SAR_{exp}^{LRT}(\eta = 1\ mPa \cdot s)}$ (%)
MFO	1	123.3	2.8	121.5	100
	2.3	112.9	2.8	111.1	91
	4.1	108.7	2.8	106.9	88
	6.3	110.8	2.8	108	89
	8.2	106.6	2.8	103.6	85
CFO	1	35.5	3.1	32.4	100
	2.1	27.2	3.1	24.1	74
	4.4	23	3.1	19.9	61
	6.1	20.3	3.1	17.2	53
	8.3	19.2	3.1	16.1	50

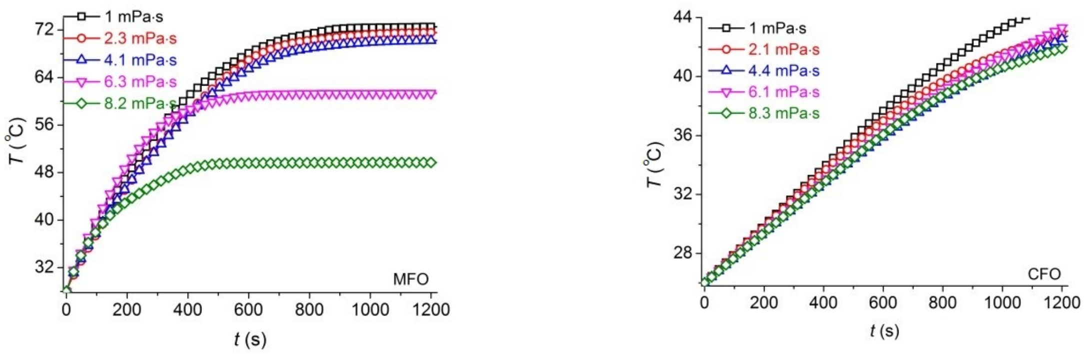

(**a**) (**b**)

Figure S6. Hyperthermia curves measured at fields of frequency f = 450 kHz, H = 15.9 kA/m for **(a)** MFO and **(b)** CFO ferrofluids of various viscosities.

Table S3. Values of SAR_{exp}, SAR_{exp}^{hys}, SAR_{exp}^{LRT}, and SLP^{LRT} at 15.9 kA/m, 450 kHz

Sample	Viscosity (mPa·s)	SAR_{exp} (W/g)	SAR_{exp}^{hys} (W/g)	SAR_{exp}^{LRT} (W/g)	$\frac{SAR_{exp}^{LRT}}{SAR_{exp}^{LRT}(\eta = 1mPa \cdot s)}$ (%)
MFO	1	284.2	3.7	280.5	100
	2.3	278	3.7	274.3	98
	4.1	273.8	3.7	270.1	96
	6.3	267.5	3.7	264.8	94
	8.2	246.6	3.7	242.9	87
CFO	1	52.3	4.2	48.1	100
	2.1	50.2	4.2	46	96
	4.4	48.1	4.2	44.1	92
	6.1	48.1	4.2	43.9	91
	8.3	46	4.2	41.8	87

Supplementary Section 6: Kc versus f at η = 1 mPa·s

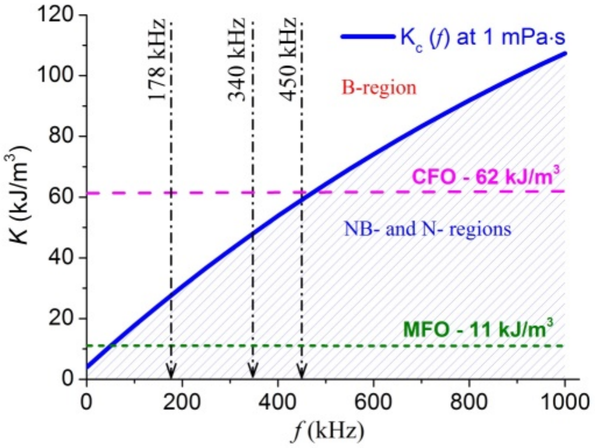

Figure S7. Illustration scheme for the MIH experiments for CFO and MFO MNPs.
